# Career perception as an upstream determinant of mental health and professional competencies in health professions education during systemic disruption

**DOI:** 10.3389/fmed.2026.1810306

**Published:** 2026-07-08

**Authors:** Haoran Chen, Zhenxing Huang, Xiaocui Huang, Zhengzhou Zhang, Mi Pan

**Affiliations:** 1 Department of Clinical Psychology, Wenzhou Seventh People’s Hospital, Wenzhou, China; 2 Department of Emergency, Zhejiang Hospital, Hangzhou, China

**Keywords:** career perception, dropout intention, medical education, mental health, pandemic

## Abstract

**Background:**

Systemic disruptions, including public health emergencies, natural disasters, and large-scale interruptions to clinical training, can destabilize health professions education and reshape students’ perceptions of their future careers. Career perception may function as a cognitive anchor that links disrupted learning environments with both psychological well-being and professional development, yet its structural role remains insufficiently understood. This study examined whether career perception operates as an upstream determinant of mental health, dropout intention, and professional competencies among medical and nursing students in a pandemic-era disrupted educational context.

**Methods:**

A cross-sectional study was conducted using secondary data from 1,707 undergraduate medical and nursing students during the pandemic. Career perception was assessed via a single-item measure of perceived change during COVID-19. Validated instruments measured empathy, lifelong learning, mental health symptoms, life satisfaction, and dropout intention. Group differences were examined using one-way and two-way ANOVA. Pearson correlation analysis assessed bivariate associations. Bayesian network analysis with bootstrap resampling was applied to explore directed probabilistic dependencies among variables.

**Results:**

Students with more positive career perceptions demonstrated higher levels of empathy, lifelong learning orientation, and life satisfaction, and lower levels of anxiety, depression, and dropout intention (all *p* < 0.001). Career perception was significantly associated with all major outcomes (|*r*| = 0.12–0.41). Bayesian network analysis identified career perception as an upstream node with robust directed effects on dropout intention (arc strength = 1.00) and lifelong learning (arc strength = 0.89). Dropout intention emerged as a central mediator linking career perception to depression, anxiety, and satisfaction. Lifelong learning and satisfaction, in turn, directly influenced empathy. These structural relationships were highly stable across bootstrap samples.

**Conclusion:**

In a disrupted health professions education context, career perception functioned as an upstream determinant shaping both mental health and professional competencies. The findings suggest that professional meaning-making and career reflection should be treated as core educational support during periods of systemic instability.

## 1 Introduction

Medical education extends beyond the transmission of biomedical knowledge and clinical competencies; it represents a formative developmental process through which students progressively construct their professional identity as future physicians (1). Yet medical education is not insulated from social disruption. A wide range of events—including public health emergencies, infectious disease outbreaks, natural disasters, climate-related events, geopolitical instability, or sudden interruptions to clinical services—can destabilize the conditions under which students learn to become clinicians (2). For medical and nursing students, such events affect delivery more than timetable. They may reduce patient contact, suspend or shorten clerkships, weaken mentoring relationships, disrupt peer learning, and make academic progression less predictable. Recent research that studied health worker education during COVID-19 showed how quickly training systems can be reorganized under crisis conditions, with long-term lessons for educational resilience and policy design (3, 4). The point is broader than the pandemic itself: when the learning environment is destabilized, students must continue constructing professional commitments while the usual sources of clinical socialization are partly withdrawn. Career perception—defined as students’ cognitive and affective appraisal of the medical profession, encompassing its perceived value, demands, risks, and personal significance—constitutes a foundational element in the cultivation of core professional qualities, including empathy, ethical commitment, and an orientation toward lifelong learning (5, 6). During stable training, this trait is shaped through repeated contact with patients, role models, teachers, and peers. During disruption, the trait may change more abruptly. Students may see healthcare work as more meaningful and socially necessary, or they may become more aware of occupational risk, workload, uncertainty, and personal cost. Recent medical education scholarship has increasingly argued that professional identity cannot be understood apart from social and structural context (7). This matters for the present study because disruptions place students at the intersection of two forces. On one side, crises can intensify the visibility of healthcare workers and make professional responsibility more salient. On the other side, the same crises can weaken the educational experiences that normally support belonging, competence, and professional confidence. Evidence from pandemic-era online education also suggests that reduced interaction with patients, faculty, and clinical teams can affect professional identity formation among medical students (8). The educational question, then, is not only whether a crisis increases distress. It is whether students’ changing appraisal of the profession helps organize the pathway from disrupted training to psychological and professional outcomes.

COVID-19 therefore provides a useful empirical setting for examining a more general educational mechanism (9, 10). During the pandemic, frontline healthcare professionals became highly visible during the public health crisis (11, 12). Although most medical students were not directly involved in frontline clinical care, they were nonetheless extensively exposed to these narratives through institutional communication, media discourse, and personal observation. Empirical studies across diverse national contexts have shown that such heightened professional visibility substantially shaped students’ career-related cognitions, with some reporting strengthened professional calling and commitment, while others experienced increased fear, uncertainty, and emotional burden regarding their future careers (12–14). Large-scale surveys further indicate that concerns related to personal safety, future working conditions, and long-term professional sustainability became salient components of occupational appraisal during this period (15). On the other hand, the prolonged and widespread shift to online teaching fundamentally disrupted the structure of medical education (16). In response to infection-control measures, traditional face-to-face instruction and clinical clerkships were rapidly replaced by distance-learning modalities, leading to substantial reductions in direct patient contact, peer interaction, and experiential learning opportunities that are central to professional socialization and identity formation (17, 18). Accumulating evidence from systematic reviews and multinational studies indicates that sustained reliance on online learning was associated with increased social isolation, diminished academic engagement, reduced perceived learning effectiveness, and a weakened sense of professional belonging among medical students (19). Moreover, pandemic-era research consistently linked these educational disruptions to elevated levels of anxiety, depressive symptoms, and burnout, alongside less favorable career outlooks and reduced learning motivation (20, 21). The value of studying pandemic-era data in 2026 therefore lies not only in documenting a historical event, but also in understanding a recurring educational problem: how students’ professional meaning-making operates when health professions education is placed under systemic strain. This issue is also relevant to Chinese medical education. During the COVID-19 period, Chinese medical students experienced prolonged online learning, disruptions to clinical training, and uncertainty regarding professional development. Consequently, understanding how career perception relates to psychological well-being and professional competencies during periods of educational instability may provide useful insights for supporting medical students in China and strengthening preparedness for future disruptions.

Despite the rapidly expanding literature on medical students’ experiences during the COVID-19 pandemic, much of the existing research remains predominantly descriptive, focusing on prevalence estimates of anxiety, depression, burnout, and stress (22–24). While numerous cross-sectional surveys have documented heightened psychological distress during this period, relatively few studies have moved beyond symptom quantification to investigate the psychological mechanisms through which abrupt changes in occupational and educational environments are internalized. In particular, limited attention has been directed toward understanding how pandemic-related disruptions reshape career perception and professional meaning-making, or how such shifts subsequently influence the development of core professional competencies. From a pedagogical and theoretical standpoint, the pandemic can be conceptualized as a quasi-natural educational context that simultaneously amplified occupational salience and uncertainty: medical students were confronted with heightened awareness of professional risk and responsibility while experiencing reduced structure, feedback, and social reinforcement within their learning environments.

Therefore, this study aimed to examine career perception as an upstream determinant of mental health and professional competencies among medical and nursing students in a disrupted educational context. Specifically, we sought to: (1) examine the relationships between career perception and mental health indicators, including anxiety, depression, and life satisfaction; (2) investigate whether dropout intention functions as an intermediary pathway linking career perception to psychological outcomes; and (3) explore how career perception relates to professional competencies, including empathy and lifelong learning. By situating pandemic-era data within the broader problem of systemic disruption in health professions education, this study seeks to inform educational strategies that support professional meaning-making, psychological well-being, and sustained professional development.

## 2 Method

### 2.1 Setting and participants

The present analysis used data from a previously conducted cross-sectional, online survey of undergraduate medical and nursing students enrolled in four universities (25). The survey was administered during the COVID-19 period, when universities had shifted to remote (non–face-to-face) teaching. Students who agreed to participate provided electronic informed consent and then completed an anonymous web-based questionnaire. For the publicly available validated dataset, only fully completed questionnaires were retained. Prior to public release, the original investigators anonymized all records and removed any personally identifiable information. The current study represents a secondary analysis of these de-identified data and involved no new participant contact; ethical review was obtained from the Ethics Committee of Wenzhou Seventh People’s Hospital (approval number: EC-20260129). The original survey protocol had been approved by the relevant institutional ethics committees (approval numbers: CEImLAR-PI-440 and 03-CIEI-UNA-PUNO).

### 2.2 Procedure

Data was gathered through a cross-sectional design using a self-administered online questionnaire. Recruitment and survey administration were implemented via the SurveyMonkey® platform between August 2020 and April 16, 2021 (25). Invitations were distributed by email and included an information sheet describing the study aims and procedures, emphasizing voluntary participation and the option to discontinue at any time. To reduce the risk of repeated submissions, the survey was configured to limit participation to a single response per access route. After data collection, the dataset underwent quality control procedures, including verification of respondents’ location using IP-based geolocation and exclusion of questionnaires that were incomplete.

### 2.3 Measurement

#### 2.3.1 Demographic information

Participants completed a structured self-administered questionnaire assessing age, sex, career perception, and dropout intention. Age (years) was analyzed as a continuous variable, and sex (male/female) was treated as a categorical variable. Career perception was assessed by asking whether participants’ perception of their career choice had changed during the pandemic, with three response options: “the same,” “better,” or “worse.” Dropout intention was measured by asking how often participants had considered dropping out, rated on a five-point Likert scale from 1 (never) to 5 (always), with higher scores indicating stronger dropout intention.

#### 2.3.2 Empathy

Empathy was measured using the 20-item Jefferson Scale of Empathy (JSE), which is commonly applied in samples of medical and other health-professions students (26). The JSE captures empathy as a predominantly cognitive, patient-oriented capacity—namely, understanding patients’ experiences, concerns, and perspectives and conveying that understanding in a helpful manner. Items are scored on a 7-point Likert scale from 1 (strongly disagree) to 7 (strongly agree), with higher scores indicating greater empathy. In this study, the JSE demonstrated good internal consistency (Cronbach’s α = 0.80).

#### 2.3.3 Lifelong learning

Lifelong learning was evaluated using the Jefferson Scale of Physician Lifelong Learning (JeffSPLL) (27). The instrument contains 14 items, each rated on a 4-point Likert scale from 1 (strongly disagree) to 4 (strongly agree). Item scores are summed to create a total score, with higher scores indicating a stronger orientation toward lifelong learning. In the current sample, the JeffSPLL demonstrated good internal consistency (Cronbach’s α = 0.84).

#### 2.3.4 Mental health

Depressive and anxiety symptoms were assessed using the Patient Health Questionnaire-2 (PHQ-2) (28) and the Generalized Anxiety Disorder-2 (GAD-2) (29), respectively. Both instruments are brief screening measures consisting of two items rated on a 4-point Likert scale from 0 (not at all) to 3 (nearly every day), reflecting symptom frequency over the past 2 weeks. Item scores are summed to yield total scores ranging from 0 to 6, with higher scores indicating greater symptom severity. In the present study, internal consistency was acceptable for the PHQ-2 and GAD-2 (Cronbach’s α = 0.77 and 0.85, respectively).

#### 2.3.5 Satisfaction

Life satisfaction was assessed using the Satisfaction with Life Scale (SWLS) (30). The SWLS is a 5-item measure that evaluates individuals’ global cognitive judgments of their life satisfaction. Each item is rated on a 7-point Likert scale from 1 (strongly disagree) to 7 (strongly agree), with total scores calculated by summing item responses; higher scores indicate greater life satisfaction. In the present study, the SWLS demonstrated good internal consistency (Cronbach’s α = 0.86).

### 2.4 Statistical analysis

Data analyses were performed using SPSS (Version 26.0) and R software (Version 4.2.0). Continuous variables were summarized as means with standard deviations (SD), and categorical variables were presented as frequencies and percentages. Descriptive statistics were first computed to characterize the demographic profile of the 1,707 participants and to examine the distributional properties of all study variables. Pearson correlation analyses were then conducted to assess bivariate associations among the key variables of interest. To examine differences in psychological and professional outcomes across the three levels of career perception, one-way analyses of variance (ANOVA) were performed, followed by Tukey-adjusted *post hoc* tests for pairwise comparisons where appropriate. In addition, two-way ANOVA models were applied to test potential interaction effects between career perception and gender. Effect sizes for group differences were quantified using partial eta-squared (*η*^2^*p*).

To further investigate the directed probabilistic dependencies among study variables, Bayesian network analysis was conducted using a score-based structure-learning approach based on the hill-climbing algorithm. Network stability was assessed via nonparametric bootstrap resampling with 2,000 iterations. Edge robustness was evaluated using two complementary indices: arc strength, defined as the proportion of bootstrap samples in which a given edge was present, and directional probability, defined as the proportion of bootstrap samples supporting a specific edge direction. Only edges meeting predefined robustness criteria (arc strength > 0.80 and directional probability > 0.50) were retained in the final network. For the resulting structure, standardized path coefficients (β) were estimated from local conditional models by regressing each node on its parent nodes and standardizing the corresponding coefficients, thereby quantifying the magnitude of probabilistic dependencies. All statistical tests were two-tailed, with a significance threshold set at the 5% alpha error level (*p* < 0.05).

## 3 Results

### 3.1 Descriptive statistics and intercorrelations

The demographic characteristics of the participants and the descriptive statistics for the study variables are summarized in Table 1. A total of 1,707 participants were included in the final analysis. The sample was predominantly female (*n* = 1,266, 74.17%), with males representing 25.83% (*n* = 441) of the cohort. The mean age of the participants was 21.42 years (SD = 3.31). The study population comprised 1,107 medical students (64.85%) and 600 nursing students (35.15%). Regarding career perception, nearly half of the respondents perceived their career prospects as “Better” (*n* = 848, 49.68%), while 33.92% (*n* = 579) felt they remained the “Same,” and a minority (*n* = 280, 16.40%) viewed them as “Worse.” The scores for empathy, lifelong learning, and satisfaction were 114.15 ± 12.85, 45.54 ± 5.86, and 16.75 ± 4.75, respectively. Regarding mental health indicators, the mean scores for Anxiety and Depression were 2.19 and 2.10 and the overall level of dropout intention was 1.73. Pearson correlation analyses were subsequently conducted to examine the interrelationships among the key variables (Table 2). Career perception was significantly associated with all major outcomes, showing a strong positive correlation with dropout intention (*r* = 0.41, *p* < 0.001) and moderate positive correlations with depression (*r* = 0.28, *p* < 0.001) and anxiety (*r* = 0.22, *p* < 0.001). In contrast, more positive career perception was associated with higher levels of lifelong learning (*r* = −0.28, *p* < 0.001), satisfaction (*r* = −0.29, *p* < 0.001), and empathy (*r* = −0.12, *p* < 0.001). Regarding demographic variables, age showed negative correlations with empathy, satisfaction, anxiety, and depression. Gender showed correlations with career perception, empathy, and anxiety.

**Table 1:** Demographic characteristics and descriptive statistics of study variables (*n* = 1,707).

Variables	Categories	*M*	*SD*	*N*	*P*ercentage
Age		21.42	3.31		
Gender					
	Male			441	25.83%
	Female			1,266	74.17%
Discipline					
	Medicine			1,107	64.85%
	Nursing			600	35.14%
Career perception					
	Better			848	49.68%
	Same			579	33.92%
	Worse			280	16.40%
Empathy		114.15	12.85		
Lifelong learning		45.54	5.86		
Satisfaction		16.75	4.75		
Anxiety		2.19	1.70		
Depression		2.10	1.58		
Drop out		1.73	0.97		

**Table 2:** Intercorrelations among variables used in this study.

Variables	1	2	3	4	5	6	7
1. Career perception	1						
2. Empathy	−0.12^***^	1					
3. Lifelong learning	−0.28^***^	0.29^***^	1				
4. Satisfaction	−0.29^***^	0.18^***^	0.32^***^	1			
5. Anxiety	0.22^***^	−0.01	−0.10^***^	−0.34^***^	1		
6. Depression	0.28^***^	−0.05^*^	−0.14^***^	−0.40^***^	0.65^***^	1	
7. Drop out	0.41^***^	−0.10^***^	−0.25^***^	−0.38^***^	0.40^***^	0.43^***^	1
Age	0.02	−0.09^***^	0.01	−0.05^*^	−0.11^***^	−0.12^***^	−0.03
Gender	−0.09^***^	0.11^***^	0.02	0.01	0.10^***^	0.04	0.01

^*^p < 0.05, ^***^p < 0.001.

### 3.2 Impact of career perception on professional and mental health outcomes

**Figure 1:**
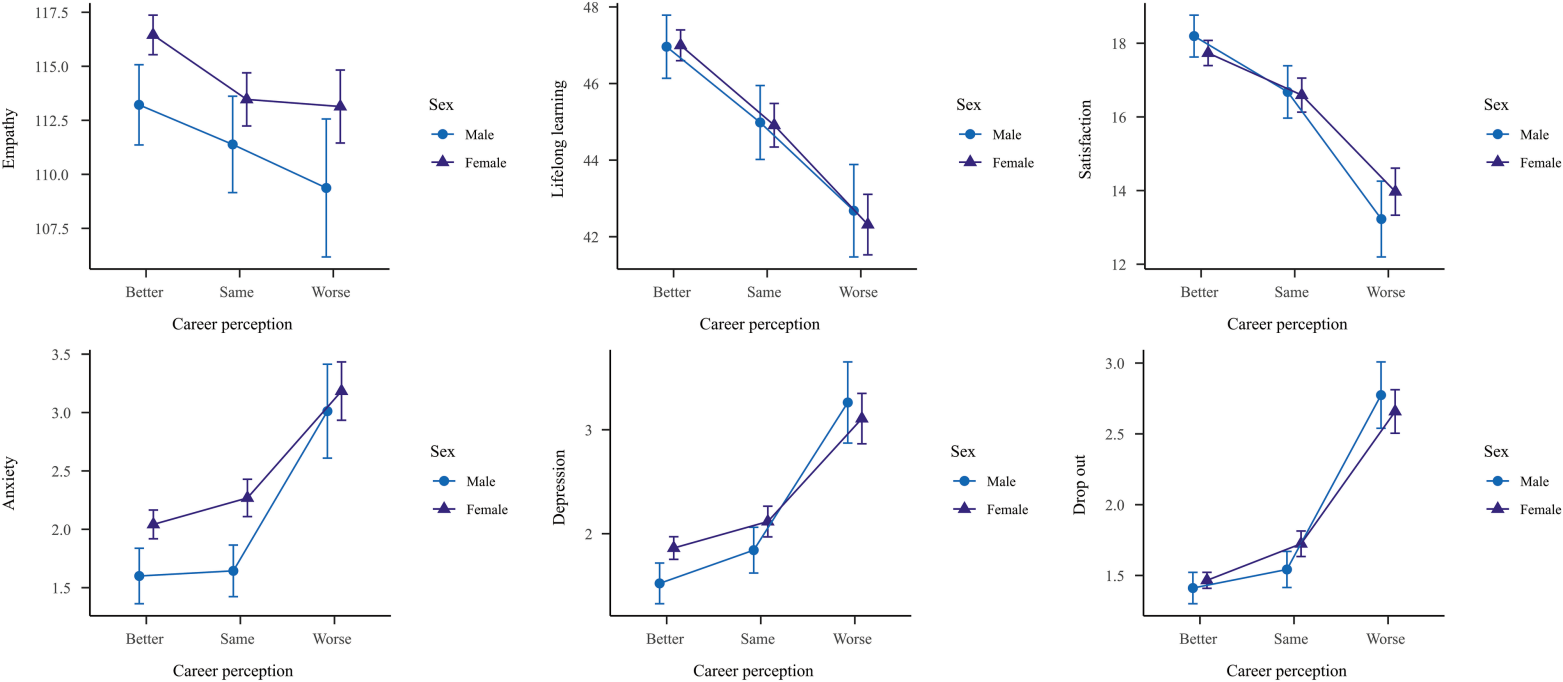
Comparison of study variables across career perception levels stratified by gender.

As illustrated in Table 3, one-way ANOVAs revealed significant differences across levels of career perception for all study variables. Students reporting a better career perception demonstrated higher levels of empathy (*F* = 13.86, *η*^2^*p* = 0.02), lifelong learning (*F* = 74.44, *η*^2^*p* = 0.08), and satisfaction (*F* = 85.86, *η*^2^*p* = 0.09) compared with those reporting the same or worse perceptions. Post-hoc analyses indicated that the better group scored significantly higher than both the same and worse groups on these professional-related outcomes (all *p* < 0.001), while the same group also outperformed the worse group on lifelong learning and satisfaction (*p* ≤ 0.001). In contrast, anxiety (*F* = 55.92, *η*^2^*p* = 0.06), depression (*F* = 87.21, *η*^2^*p* = 0.09), and dropout intention (*F* = 218.59, *η*^2^*p* = 0.20) increased progressively as career perception worsened. Furthermore, two-way ANOVAs were used to test the interaction between career perception and sex which showed no significant interaction effects on empathy (*F* = 0.44, *p* = 0.64), lifelong learning (*F* = 0.11, *p* = 0.90), satisfaction (*F* = 1.46, *p* = 0.23), anxiety (*F* = 1.53, *p* = 0.22), depression (*F* = 2.37, *p* = 0.09) and drop out intention (*F* = 2.41, *p* = 0.09) (Figure 1).

**Table 3:** Comparison of study variables across different career perception groups with *post-hoc*.

Variables	Career perception			*F*	*η* ^2^ *p*	*Post-hoc* (*p*)
	Better	Same	Worse			
Empathy	115.77 ± 12.24	112.83 ± 13.38	112.01 ± 12.95	13.86	0.02	*B > S: < 0.001* *B > W: < 0.001* *S vs W: ns*
Lifelong learning	46.99 ± 5.36	44.93 ± 6.02	42.43 ± 5.58	74.44	0.08	*B > S: < 0.001* *B > W: < 0.001* *S > W: 0.001*
Satisfaction	17.83 ± 4.37	16.62 ± 4.73	13.75 ± 4.60	85.86	0.09	*B > S: < 0.001* *B > W: < 0.001* *S > W: < 0.001*
Anxiety	1.95 ± 1.63	2.08 ± 1.61	3.13 ± 1.80	55.92	0.06	*B < S: ns* *B < W: < 0.001* *S < W: < 0.001*
Depression	1.79 ± 1.42	2.03 ± 1.50	3.15 ± 1.74	87.21	0.09	*B < S: 0.008* *B < W: < 0.001* *S < W: < 0.001*
Drop out	1.45 ± 0.74	1.67 ± 0.90	2.69 ± 1.09	218.59	0.20	*B < S: < 0.001* *B < W: < 0.001* *S < W: < 0.001*

### 3.3 Bayesian network analysis and structural dependencies

**Figure 2:**
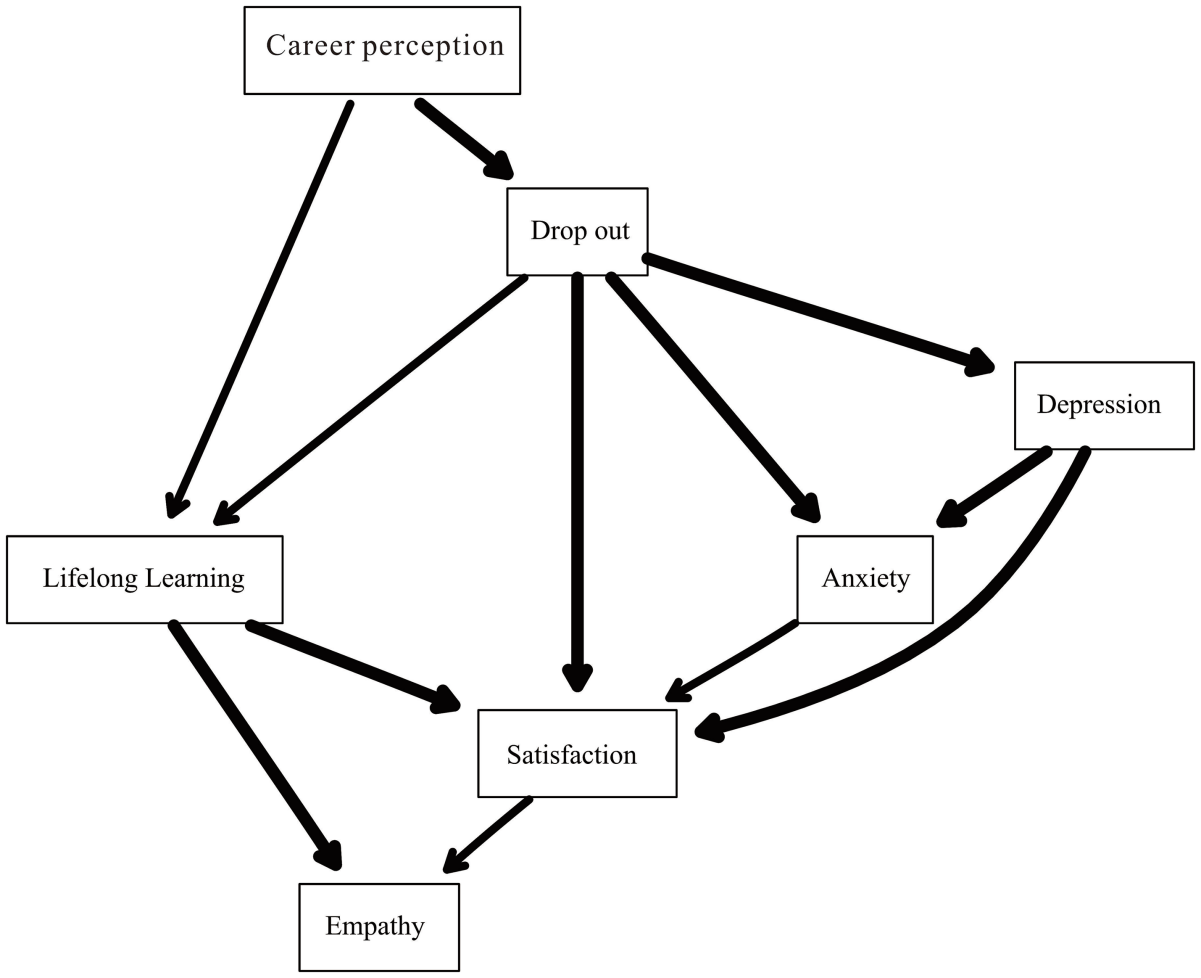
Directed acyclic graph representing the structural dependencies among study variables. Thicker arrows indicate higher arc strength.

**Figure 3:**
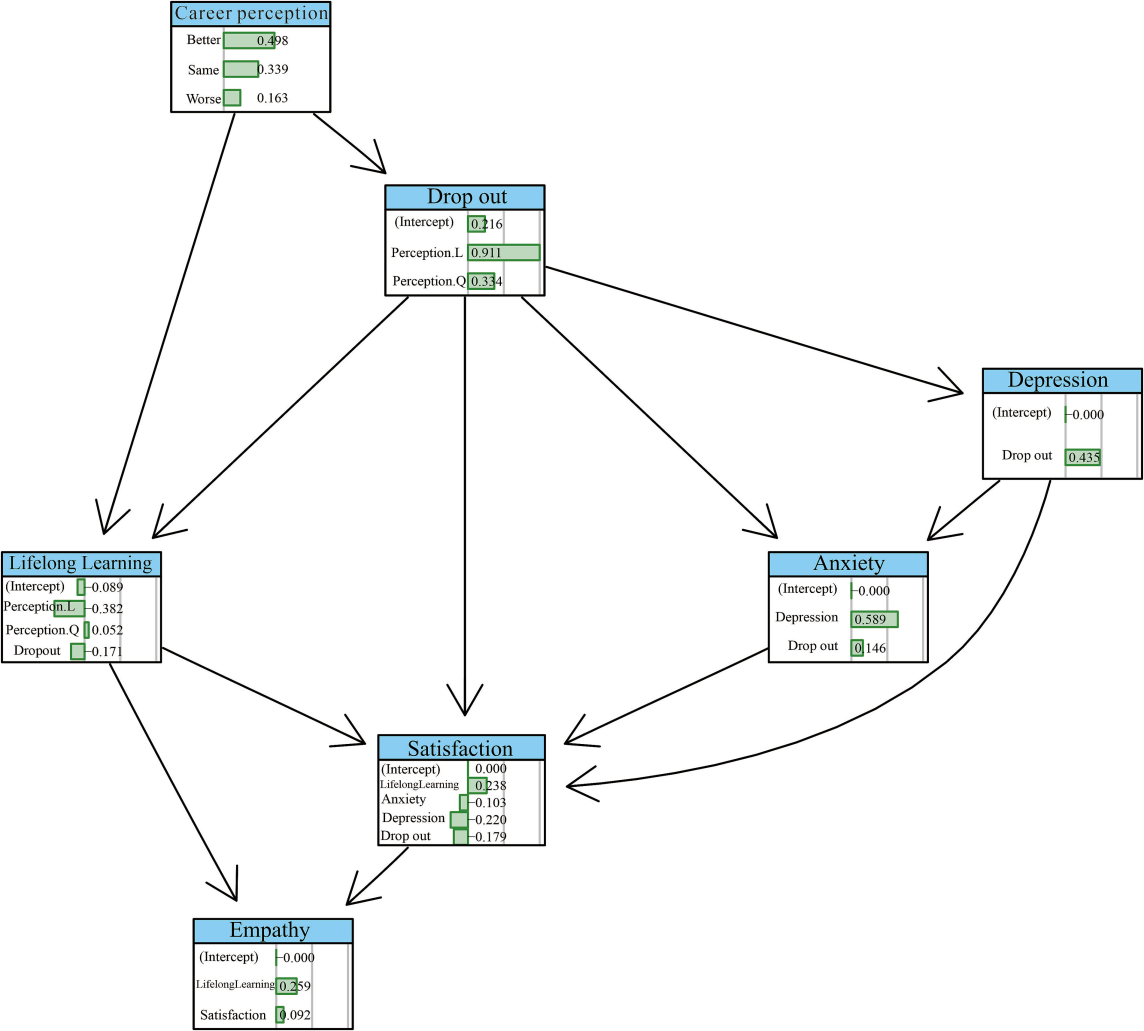
Bayesian network structure with standardized path coefficients (β) illustrating the magnitude of probabilistic dependencies.

Bayesian Network analysis was conducted to explore the directed dependencies and the complex structural relationships among variables. The network structure was learned using a bootstrap-based Bayesian structure learning approach, and edge robustness was evaluated using arc strength and directional probability (Table 4; Figure 2). The resulting network identified career perception as an upstream node, showing a highly stable directed association with dropout intention (arc strength = 1.00; directional probability = 1.00) and a strong direct connection with lifelong learning (arc strength = 0.89; directional probability = 1.00). Importantly, the Bayesian network further revealed that the influence of career perception on a broader set of outcomes was largely transmitted through dropout intention, which emerged as a central intermediary node. Dropout intention showed robust outgoing connections to satisfaction, anxiety, and depression (arc frequencies = 0.99–1.00; directional probabilities = 0.75–0.97). Empathy appeared as the most downstream node in the network directly influenced by Lifelong learning (arc strength = 1.00; directional probability = 0.95) and Satisfaction (arc strength = 0.86; directional probability = 0.56). Parameter estimates from Figure 3 indicate that dropout intention was positively influenced by career perception (coefficient = 0.91) and, in turn, exerted a direct effect on depression (coefficient = 0.44) and satisfaction (coefficient = 0.18). Anxiety and depression were closely linked, with depression exerting a strong influence on anxiety (coefficient = 0.59). Satisfaction was influenced by a convergence of factors, including lifelong learning (0.24), dropout intention (0.18), anxiety (−0.10), and depression (−0.220). Finally, empathy appeared as the most downstream node in the network. It was directly influenced by lifelong learning (coefficient = 0.26) and satisfaction (coefficient = 0.09).

**Table 4:** Arc strength, arc direction, and beta coefficients for the Bayesian networks.

From	To	Arc strength	Arc direction
Career perception	Lifelong learning	0.89	1.00
Career perception	Drop out	1.00	1.00
Drop out	Lifelong learning	0.84	0.56
Drop out	Satisfaction	1.00	0.95
Drop out	Anxiety	0.99	0.97
Drop out	Depression	1.00	0.75
Depression	Satisfaction	0.99	0.71
Depression	Anxiety	1.00	0.65
Lifelong learning	Empathy	1.00	0.95
Lifelong learning	Satisfaction	1.00	0.78
Anxiety	Satisfaction	0.81	0.77
Satisfaction	Empathy	0.86	0.56

Arc strength refers to the proportion of times that an arc appeared in the bootstrap samples, regardless of direction. Arc direction refers to the proportion of times that the arc pointed in that given direction.

## 4 Discussion

The present study provides an integrative examination of how medical and nursing students’ career perception during the COVID-19 pandemic relates to professional competencies and mental health outcomes. By combining group comparisons with Bayesian network analysis, this study moves beyond descriptive associations and offers a probabilistic structural interpretation of how career perception function as an upstream determinant shaping students’ psychological well-being and professional development. Three key findings merit particular attention.

### 4.1 Career perception as an upstream determinant of professional and mental health outcomes

The primary finding of this study is that career perception functions as a foundational upstream factor that initiates a cascade of professional and psychological outcomes. At the descriptive level, students who reported a more positive career perception consistently demonstrated higher levels of empathy, stronger lifelong learning orientation, and greater life satisfaction, alongside lower levels of anxiety, depressive symptoms, and dropout intention. These patterns are broadly consistent with prior research indicating that positive career perception is closely linked to academic engagement, resilience, and psychological well-being among health professions trainees (31, 32). Importantly, the Bayesian network analysis extends these findings by suggesting a directional structure in which students’ cognitive and affective appraisal of the medical profession exerts a direct influence on both professional commitment and subsequent mental health outcomes. During the pandemic, medical and nursing students were simultaneously confronted with intensified societal visibility of the healthcare profession—marked by heightened occupational risk, moral responsibility, and public expectation—and substantial disruption to traditional educational structures, including reduced clinical exposure, limited peer interaction, and prolonged reliance on online learning (33). Within this context of uncertainty and constraint, career perception have assumed heightened salience as a cognitive anchor through which students interpreted external stressors, regulated motivation, and evaluated the personal costs and benefits of continuing their training. From a theoretical standpoint, these findings are highly consistent with social cognitive career theory, which emphasizes the central role of career-related value appraisals and outcome expectations in shaping persistence, self-regulation, and emotional responses, particularly under conditions of environmental instability (34). In the pandemic context, students with a more positive career perception have been better positioned to construe heightened professional risks and educational disruptions as meaningful challenges aligned with long-term vocational goals, thereby sustaining learning engagement and buffering against psychological distress (35). In contrast, negative or uncertain career perception—when compounded by diminished experiential learning opportunities and weakened social reinforcement, have undermined perceived goal attainability and professional self-efficacy, increasing vulnerability to disengagement, dropout intention, and emotional distress (36). The Bayesian network findings provide empirical support for this interpretation by positioning career perception as a highly stable upstream node with robust directed effects on dropout intention and lifelong learning, rather than as a downstream consequence of anxiety or depressive symptoms. Taken together, these results suggest that during the epidemic period, career perception functioned not merely as an attitudinal byproduct of psychological distress, but as a central regulatory mechanism linking pandemic-related educational disruption to both mental health outcomes and the development of core professional competencies.

### 4.2 Dropout intention as a pandemic-sensitive psychological mediator

A second key contribution of this study lies in identifying dropout intention as a pandemic-sensitive psychological mediator linking career perception to mental health and well-being outcomes. Although dropout intention is commonly conceptualized as a distal behavioral endpoint in medical education research, the present Bayesian network analysis positions it as a central intermediary psychological state through which career-related cognition is translated into emotional and evaluative outcomes. Specifically, career perception exhibited a highly robust directed association with dropout intention, which in turn exerted stable downstream effects on depressive symptoms, anxiety, and life satisfaction. This structural pattern suggests that, during the epidemic period, contemplating withdrawal from training reflected an active process of psychological appraisal rather than a passive reaction to emotional distress. The prominence of dropout intention as a mediating mechanism is particularly intelligible within the context of the COVID-19 pandemic. The abrupt transition to online instruction, suspension or curtailment of clinical clerkships, and prolonged uncertainty regarding academic progression fundamentally altered students’ expectations of medical training and professional development (37). Concurrently, intensified media exposure to physician burnout, occupational risk, and moral injury have heightened students’ cost–benefit evaluations of a future medical career. Under these conditions, dropout intention have emerged as a cognitively accessible coping-related appraisal, signaling a perceived misalignment between personal resources and anticipated professional demands (38). Consistent with stress appraisal and motivational frameworks, perceptions of threatened goal attainability and diminished controllability are more likely to precede emotional exhaustion, depressive affect, and anxiety than to merely co-occur with them (39). Importantly, the present findings indicate that the psychological impact of dropout intention extends beyond negative affect to encompass reduced life satisfaction, thereby influencing both effective and evaluative components of well-being. This pattern supports the interpretation of dropout intention as a broader form of motivational disruption, characterized by weakened future orientation and erosion of professional commitment. In the pandemic context—where external structures that typically sustain persistence, such as face-to-face mentorship, peer interaction, and clinical role modeling, were substantially weakened—such motivational disruptions have been particularly consequential (40). Students lacking a stable and positive career perception have been more inclined to interpret educational disruptions as evidence of an unsustainable professional trajectory, thereby amplifying dropout-related cognitions and their emotional sequelae. Taken together, these findings suggest that dropout intention should not be viewed solely as a terminal educational outcome, but rather as an early warning signal of compromised professional meaning-making and heightened emotional vulnerability during periods of systemic disruption.

### 4.3 Professional growth pathways: lifelong learning, satisfaction, and empathy

Beyond mental health outcomes, the present findings indicate that professional growth during the pandemic followed a pathway in which lifelong learning orientation and satisfaction played a central role in shaping empathy. Within the Bayesian network, lifelong learning emerged as a key downstream consequence of career perception and dropout intention, whereas empathy appeared as the most distal professional outcome, influenced primarily by lifelong learning and satisfaction rather than directly by anxiety or depressive symptoms. This structural pattern is consistent with prior evidence suggesting that empathy among medical trainees is closely linked to sustained learning engagement and reflective capacity, rather than being determined solely by emotional well-being (41). Empirical research has consistently demonstrated that students with a stronger orientation toward lifelong learning are more inclined to engage in reflective practice, actively seek feedback, and integrate patient perspectives into their clinical reasoning, all of which contribute to the development and maintenance of empathic competence (42). In parallel, higher levels of academic and life satisfaction have been associated with greater professional engagement, stronger identification with the medical role, and more patient-centered attitudes among medical students. Within the context of the COVID-19 pandemic—when clinical exposure, bedside learning, and interpersonal interaction were substantially constrained (4, 43)—maintaining lifelong learning orientation and satisfaction with training may have become increasingly important for supporting empathy and professional development. Under such conditions, empathy appears less as a direct emotional response to stress and more as an emergent professional capacity fostered through ongoing learning engagement and positive appraisal of the training experience.

## 5 Conclusion

This study demonstrates that career perception plays a central role in shaping both professional development and mental health among medical and nursing students during the COVID-19 pandemic. By integrating group comparisons with Bayesian network analysis, the findings indicate that career perception functions as an upstream determinant, influencing dropout intention and lifelong learning orientation, which in turn affect psychological well-being, satisfaction, and empathy. Dropout intention emerged as a psychologically meaningful intermediary linking negative career appraisal to anxiety, depressive symptoms, and reduced satisfaction, while professional growth followed a downstream pathway in which lifelong learning and satisfaction supported the development of empathy. These findings highlight the importance of addressing career perception and professional meaning-making as core components of medical education, particularly during periods of heightened uncertainty and educational disruption. Interventions that strengthen students’ understanding of professional value, clarify career trajectories, and support learning engagement help mitigate psychological distress while simultaneously sustaining professional competencies.

## 6 Clinical and educational implications

This study highlights several practical implications for medical and nursing education, particularly during periods of educational disruption. First, career perception should be recognized as a core educational and clinical concern. Structured opportunities for career reflection and professional meaning-making help stabilize students’ motivation and psychological well-being when training conditions are uncertain (44). Second, dropout intention appears to function as an early psychological indicator of vulnerability rather than merely a risk of academic attrition (45). Routine attention to dropout-related thoughts, alongside symptoms of anxiety and depression, may support earlier identification of at-risk students and guide timely intervention focused on clarifying training pathways and reinforcing professional purpose. Finally, sustaining lifelong learning orientation and academic satisfaction are critical for preserving empathy and professional development when clinical exposure is limited (46, 47). Educational strategies that promote self-directed learning, reflective practice, and supportive mentorship may help integrate mental health support with professional training and strengthen students’ resilience during periods of crisis.

## 7 Strengths and limitations

Several strengths and limitations should be acknowledged. A major strength of this study is the relatively large sample of 1,707, which provided sufficient variability to examine professional and psychological outcomes across levels of career perception. Methodologically, the integration of group comparisons, correlation analysis, Bayesian network structure learning, and bootstrap resampling allowed the study to move beyond descriptive prevalence estimates and examine the stability of directed probabilistic dependencies among variables. The study also has limitations. First, the cross-sectional design precludes definitive causal inference. Although Bayesian network analysis allowed probabilistic modeling of directed dependencies, the identified directions should be interpreted as structural tendencies rather than temporal causality. Longitudinal studies are needed to confirm whether changes in career perception precede subsequent changes in dropout intention, mental health, and professional development. Second, the study sample was limited to undergraduate medical and nursing students in university-based training programs, and the findings may not be fully generalizable to other educational systems, postgraduate trainees, or practicing healthcare professionals. Third, the study used pandemic-era secondary data rather than a newly collected China-based survey. This limits direct inference for Chinese medical schools, although it also preserves information from a disruption period that cannot be fully recreated retrospectively. Fourth, career perception was assessed using a single-item measure reflecting perceived change during the pandemic. While this item captured students’ global occupational appraisal in a pragmatic manner, it may not fully represent the multidimensional nature of career perception and professional meaning-making. Future research should use longitudinal designs, more comprehensive career perception instruments, and China-based multicenter samples to validate and extend these findings.

## Data Availability

The original contributions presented in the study are included in the article/supplementary material, further inquiries can be directed to the corresponding authors.
